# A systematic, realist review of zooprophylaxis for malaria control

**DOI:** 10.1186/s12936-015-0822-0

**Published:** 2015-08-12

**Authors:** Blánaid Donnelly, Lea Berrang-Ford, Nancy A Ross, Pascal Michel

**Affiliations:** Department of Geography, McGill University, Burnside Hall Building, 805 Sherbrooke St West, Montreal, QC H3A 0B9 Canada; Public Health Risk Sciences Division, Public Health Agency of Canada, 3200 Sicotte, PO Box 5000, Saint-Hyacinthe, QC J2S 7C6 Canada

**Keywords:** Zooprophylaxis, Zoopotentiation, Malaria, Livestock, Vector-borne disease, Integrated vector management

## Abstract

**Background:**

Integrated vector management (IVM) is recommended as a sustainable approach to malaria control. IVM consists of combining vector control methods based on scientific evidence to maximize efficacy and cost-effectiveness while minimizing negative impacts, such as insecticide resistance and environmental damage. Zooprophylaxis has been identified as a possible component of IVM as livestock may draw mosquitoes away from humans, decreasing human-vector contact and malaria transmission. It is possible, however, that livestock may actually draw mosquitoes to humans, increasing malaria transmission (zoopotentiation). The goal of this paper is to take a realist approach to a systematic review of peer-reviewed literature to understand the contexts under which zooprophylaxis or zoopotentiation occur.

**Methods:**

Three electronic databases were searched using the keywords ‘zooprophylaxis’ and ‘zoopotentiation’, and forward and backward citation tracking employed, to identify relevant articles. Only empirical, peer-reviewed articles were included. Critical appraisal was applied to articles retained for full review.

**Results:**

Twenty empirical studies met inclusion criteria after critical appraisal. A range of experimental and observational study designs were reported. Outcome measures included human malaria infection and mosquito feeding behaviour. Two key factors were consistently associated with zooprophylaxis and zoopotentiation: the characteristics of the local mosquito vector, and the location of livestock relative to human sleeping quarters. These associations were modified by the use of bed nets and socio-economic factors.

**Discussion:**

This review suggests that malaria risk is reduced (zooprophylaxis) in areas where predominant mosquito species do not prefer human hosts, where livestock are kept at a distance from human sleeping quarters at night, and where mosquito nets or other protective measures are used. Zoopotentiation occurs where livestock are housed within or near human sleeping quarters at night and where mosquito species prefer human hosts.

**Conclusion:**

The evidence suggests that zooprophylaxis could be part of an effective strategy to reduce malaria transmission under specific ecological and geographical conditions. The current scientific evidence base is inconclusive on understanding the role of socio-economic factors, optimal distance between livestock and human sleeping quarters, and the effect of animal species and number on zooprophylaxis.

**Electronic supplementary material:**

The online version of this article (doi:10.1186/s12936-015-0822-0) contains supplementary material, which is available to authorized users.

## Background

Despite renewed commitments and control efforts in recent years [1–3] malaria continues to be a major contributor to global health burden, with approximately 165 million cases in 2013 [4]. Integrated vector management (IVM) has been promoted as a sustainable approach to combat malaria [5, 6] in the face of increasing insecticide resistance of malaria vectors, and environmental and health concerns [5, 7]. This strategy involves combining chemical and non-chemical interventions targeted to specific ecological settings in a way that maximizes efficacy while minimizing cost and negative environmental impacts [5]. IVM makes use of environmental modification, environmental manipulation, chemical control methods, and biological methods [5] (Table 1).

**Table 1:** Summary of integrated vector management (IVM) approach [9, 20, 53]

Integrated vector management approach					
Method	Chemical	Biological	Environmental management		
Definition	Reduce the vector population by killing larvae or adult vectors with insecticides (e.g., DDT)	Using natural predators or pathogens of vector species	Disrupting vectoral habitat to reduce human-vector interaction and/or vector reproduction and survival		
Sub-type	–	–	Modification of human habitats and behaviours	Environmental manipulation	Environmental modification
Sub-type definition	–	–	Locating human habitats and changing behaviour to reduce vector-host contact	Long term change to physical environment to prevent vector habitats	Temporary change to physical environment to prevent larval development
Examples	Indoor residual spraying Space spraying	Larvivorous fish Nematodes Bacteria	Sleeping under bed nets Placing human settlements away from vector breeding sites Screening doors and windows Zooprophylaxis	Wetland and marsh drainage Ditch filling	Tree planting

Strategic placement of livestock sheds or pens has also been proposed as a component of IVM to reduce contact between vectors and human hosts [8, 9]. The World Health Organization (WHO) began recommending this type of intervention in 1982 as a method to divert mosquitoes from human populations [10]. This purposeful use of livestock (i.e. as dead-end hosts) to divert mosquitoes away from humans is described as active zooprophylaxis. Passive zooprophylaxis occurs where normal presence of livestock draws mosquitoes away from humans [11]. Insecticide zooprophylaxis, more commonly described in tsetse fly control, involves the use of insecticide-treated cattle and has also been investigated for the control of malaria vectors [12–15].

There remains considerable debate regarding the efficacy of zooprophylaxis [10, 11, 16–20]. In addition to the literature supporting zooprophylaxis [21, 22], there is evidence that supports zoopotentiation; livestock presence may actually *increase* malaria transmission by creating additional blood meal sources, which, in turn, can increase vector lifespan and population density [10, 11, 16]. Due to the divergent nature of the literature and the complexity of the relationship between livestock and malaria prevalence, there has been a reluctance to employ zooprophylaxis in control programmes [8, 23, 24].

The goal of this paper was to characterize and critically assess the potential for zooprophylaxis to reduce malaria transmission, with specific attention paid to the contexts under which it may be an effective component of IVM. The strategic framework for IVM calls for evidence-based decision-making in the selection of appropriate interventions that acknowledge the local context, including vector ecology, epidemiology and socio-economic factors [5].

## Methods

**Fig. 1:**
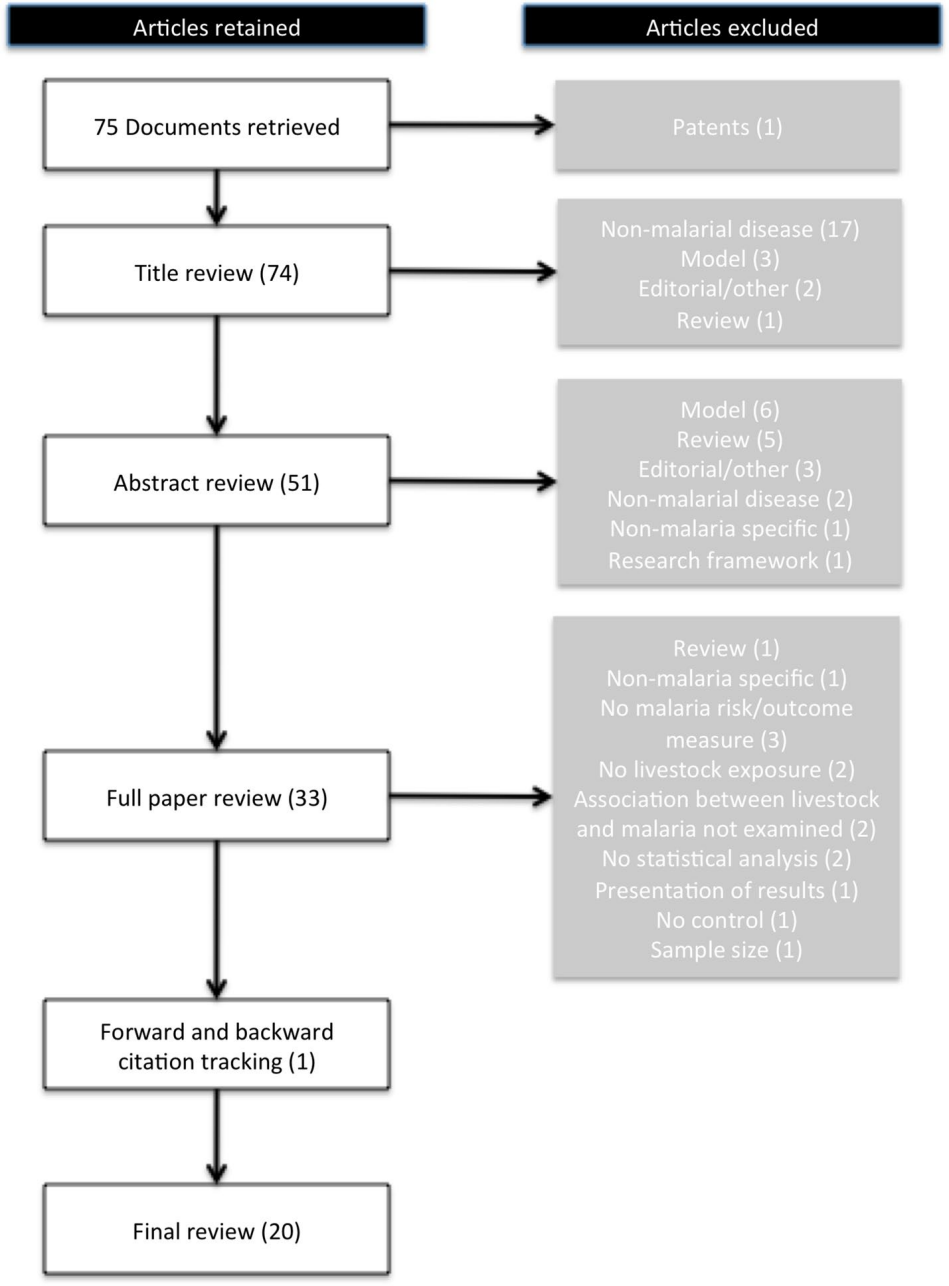
Systematic article selection process.

A modified systematic review methodology, employing realist approaches [25, 26] was applied to the self-identifying zooprophylaxis literature. This approach recognizes a priori that the scientific literature in this area is conflicting and in this case focuses on *when, why,* and *in what contexts* zooprophylaxis or zoopotentiation may occur. A meta-analysis, was not feasible due to the variety of study designs (including both observational and experimental designs) and outcome measures employed in this research area. ISI Web of knowledge, CAB Direct and PubMed databases were searched in December 2014 using the keywords ‘zooprophylaxis’ and ‘zoopotentiation’. While this invariably excluded studies of malaria risk factors that consider the presence of animals, but did not self-identify using the terms ‘zoopotentiation’ or ‘zooprophylaxis’, the search was limited to these explicit terms for two reasons: (1) to select a proxy sample of the key literature explicitly emphasizing and investigating zooprophylaxis, more likely to provide direct discussion, consideration of causal pathways of association and depth regarding the role of animals in malaria transmission; and, (2) to limit the number of results to a feasible and directly relevant sample for in-depth realist analysis. This search retrieved 75 documents after removal of duplicates. Only empirical, peer-reviewed articles that focused on either malaria infection in humans or mosquito behaviour associated with livestock presence were reviewed. Mathematical models of mosquito behaviour and review articles were excluded from the synthesis but their content was assessed to provide context for interpretation of results (Table 2). A total of 20 articles met final inclusion criteria and were retained for critical appraisal after full article review (Fig. 1). Forward and backward citation tracking were applied to the articles selected for critical appraisal with one additional relevant article identified.

**Table 2:** Inclusion and exclusion criteria for document selection

Inclusion	Exclusion
English	Non-English
Peer-reviewed articles presenting empirical research	Reviews, editorials, theoretical frameworks, mathematical models, grey literature, non-empirical studies
Considers livestock as a predictor variable	No livestock variable or comparison
Malaria risk outcome such as human biting index or diagnosed malaria infection	Malarial outcome based only on febrile illness (no confirmed diagnosis)

Data extraction from each article included author, date of publication, study location, livestock exposure, malaria risk outcome measures, study design, and study limitations. Published results reporting significant associations at the 95% confidence level were classified as supporting a significant zooprophylaxis or zoopotentiation effect. Critical appraisal [27] resulted in the exclusion of 14 articles. Reasons for exclusion (Additional file 1) were related to data analysis such as a lack of evidence of statistical significance [28, 29], and pooling of data preventing conclusions from being made on the effect of livestock on malaria risk [30]. Others were excluded based on study design issues, such as the absence of a comparison group [31] and small sample sizes [32]. The pertinent results and conclusions of each study were analysed with regard to the associations between livestock and malaria risk.

## Results

### Study characteristics

Twenty articles met inclusion criteria; 15 were observational studies and five were experimental (Table 3). Of the observational designs, there were 12 cross-sectional, two case–control, and one cohort design. The majority (16) of studies were conducted in sub-Saharan Africa (SSA), nine from East Africa, five from West Africa and two from Southern Africa. The remaining four studies were carried out in Pakistan (2), Bolivia (1) and Lao PDR (1). Two articles reported on a single study conducted in The Gambia, although each article reports analysis of a different outcome (malaria infection *versus* mosquito feeding behaviour).

**Table 3:** Summary of reviewed articles

References	Geographic location	Sample	Findings	Accounted for bed net?	Accounted for socio-economic factors?	Predominant mosquito species and characteristics (as reported by authors)	Animal-related variable	Effect
Bogh et al. [11]	The Gambia	102 pairs of children	*An. gambiae* s.s. and *An. melas*: no difference in HBI between cattle and non-cattle group. *An. arabiensis*: reduction of HBI by 30% in presence of cattle. No significant difference in sporozoite rate of all mosquito species in cattle compounds (0.97%) compared to non-cattle compounds (1.28%)	Yes	No	*An. gambiae* s.s. (72%), *An. arabiensis* (10%): relatively zoophilic, *An. melas* (18%): relatively zoophilic	Cattle present: children sleeping <20 m from at least one cow vs cattle absent: children sleeping >50 m from nearest cow (other livestock present but not considered)	Zooprophylaxis (*An. arabiensis*), none (*An. gambiae* s.s. and *An. melas*)
Bogh et al. [10]	The Gambia	102 pairs of children	No difference in parasite prevalence odds ratio between cattle and non-cattle group after adjusting for wealth. Adjusted OR 1.69 (CI 0.67–4.24), p = 0.26	Yes	Yes	As above	As above	None
Bouma and Rowland [16]	Pakistan	2,042 slides examined over 2 years	Higher parasite prevalence in children from households owning cattle (15.2%) than children without (9.5%) Mantel–Haenszel χ^2^ = 9.6, p < 0.005. Mean parasite rates and prevalence of cattle keeping were positively correlated for seven villages (r = 0.79, p = 0.036)	No	No	*Anopheles culicifaces*: zoophilic, *Anopheles stephensi*: zoophilic, *Anopheles subpictus*: zoophilic	Cattle or water buffalo kept within the household compound	Zoopotentiation
Bulterys et al. [40]	Zambia	34 case households, 27 control households	Cattle ownership was associated with reduced odds of recurrent malaria infection (adjusted OR 0.19, CI 0.05–0.69). Households with the most cattle, goats, dogs, or cats had reduced odds of recurrent infection (adjusted OR 0.13, CI 0.03–0.56)	Yes	No	*An. arabiensis*: anthropophilic/opportunistic, *An. funestus*	Animal ownership (location not measured)	Zooprophylaxis
Ghebreyesus et al. [23]	Ethiopia	2,114 children (<10 years)	Animals sleeping indoors increased the incidence rate ratio for malaria infection (adjusted RR 1.92, CI 1.29–2.85). Cattle ownership was not associated with malaria infection (1–2 cows: aRR 0.75, CI 0.39–1.45; 3–4 cows: aRR 1.18, CI 0.65–2.14; ≥5 cows: aRR 1.18, CI 0.64–2.17) nor was sheep and goat ownership (1-4 sheep/goats: aRR 0.93, CI 0.58–1.50; ≥5 sheep goats: aRR 0.81, CI 0.54–1.22)	No	Yes	*An. arabiensis*	Cattle ownership, sheep and goat ownership, animals sleep inside house	Zoopotentiation for animals sleeping indoors. No effect for sheep/goat or cattle ownership.
Habtewold et al. [12]	Ethiopia	278 mosquitoes	No significant difference in proportion of mosquitoes feeding on humans and livestock for people sleeping with livestock indoors (site B) vs livestock housed separately (site A). Higher proportion of mosquitoes feeding on cattle (93.7%) compared to humans (3.1%) for people sleeping on elevated platforms (site C) above livestock (p < 0.05). Higher proportion of cattle feeding in site C (93.7%) vs sites A (42.7%) and B (54.7%) (p < 0.001)	No	No	*An. arabiensis*: moderately zoophilic	Humans sleep in traditional houses with cattle in separate enclosures (site A), humans sleep in houses with livestock sharing dwelling at night (site B), humans sleep in tree platforms above cattle (site C)	Zooprophylaxis
Habtewold et al. [13]	Ethiopia	18 study replications, total mosquito catch not reported	No effect of untreated ox on HBC for *An. arabiensis* but ox odour increased HBC (mean catch/person/night = 22 without cattle odour, 32 with, p < 0.05). For *An. pharoensis* HBC was significantly reduced in the presence of untreated ox (catch/person/night = 50 without and 26 with, p < 0.01) but increased in presence of cattle odour (catch/person/night = 6 without and 18 with, p < 0.001). CIs included but graph printing obscures visualization for most values	NA	NA	*An. arabiensis*: zoophilic, exophagic. Secondary vector: *An. pharoensis*	“Nearby” specific distance not reported	None (*An. arabiensis*), zooprophylaxis (*An. pharoensis*)
Hadis et al. [36]	Ethiopia	611 *An. arabiensis* mosquitoes	Mosquitoes collected from mixed human-livestock dwellings had significantly lower HBI (20.2%) than mosquitoes collected from human-only dwellings (91.5%) p < 0.001	No	No	*An. arabiensis*	Human dwellings vs mixed human-cattle dwellings vs cattle sheds	Zooprophylaxis
Hewitt et al.^a^ [24]	Pakistan	643 anopheline mosquitoes	HLC increased in presence of a cow (38%, CI 8–68%), and two goats (50%, CI 16–84%)	NA	NA	*An. stephensi*: zoophilic	A cow or two goats tethered 6 m from male mosquito collectors	Zoopotentiation
Hiscox et al. [38]	Lao PDR	879 anopheline mosquitoes	Cow ownership doubled the risk of anopheline house entry (IRR 2.32, CI 1.29–4.17, p = 0.005)	Yes	Yes	*Anopheles philippinensis*	Ownership of chickens, ducks, pigs, cows, or buffaloes, and keeping large animals (pigs, cows, buffaloes below the house)	Zoopotentiation for cow ownership but no effect of owning any other animals or keeping large animals below the house
Iwashita et al. [33]	Kenya	104 houses, 1,664 anopheline mosquitoes	*An. arabiensis* abundance increased by 10% with each additional goat/sheep tethered around the house. [Exp (β) = 1.10, β = 0.10, p = 0.02]. Odds of human blood feeding were decreased 0.99 times by each goat or sheep tethered within 500 m of the household [Exp (β) = 0.99, β = −0.01, p < 0.01]	Yes	No	*An. arabiensis*: zoophilic, exophagic, *An. funestus* s.s.: anthropophilic, endophagic, *An. gambiae* s.s. anthropophagic, endophagic	Cattle or goats/sheep kept within 20 m of house	None (*An. gambiae s.s., An. funestus s.s*), zoopotentiation (*An. arabiensis*)
Lardeux et al.^a^ [54]	Bolivia	384 blood fed mosquitoes	*Anopheles pseudopunctipennis* preferred small ruminants (forage ratio 1.99, CI 1.80–2.19) to equids (1.95, CI 1.38–2.52) to humans (1.47, CI 1.25–1.69) to cows (1.15, CI 0.65–1.66) and avoided pigs (0.34, CI 0.20–0.48) and chickens (0.03, CI 0–0.75)	No	No	*An. pseudopunctipennis*: opportunistic	Various collection locations including outdoor traps and indoor resting collections	Zooprophylaxis
Maia et al.^a^ [21]	Ghana	1,017 anopheline mosquitoes	Presence of cattle reduced the number of *An. gambiae* s.s. for HLC by 66% (p < 0.0001) but increased the density of *Anopheles ziemanni* (not statistically significant). Cattle presence did not influence the HLC number from 20 m away	NA	NA	*An. gambiae* s.s.: NA, *An. ziemanni*: zoophilic	Cattle inside 6 × 7 m experimental pen	Zooprophylaxis (*An. gambiae* s.s)
Mala et al. [37]	Kenya	20 households, 417 mosquitoes	Odds of *An. arabiensis* occurrence decreased in presence of animals (OR 0.4, p = 0.03) and odds decreased with increasing distance to animal shelters (OR = 0.88, p < 0.001)	No	No	*An. arabiensis* (66%), *An. funestus* (18%), *An. pharoensis* (15%)	Presence of animals, relative distance to animal sheds	Unclear
Mutero et al. [8]	Kenya	420 households	Low malaria prevalence in irrigated villages compared to non-irrigated villages (p < 0.05). Authors attribute this to preference for cattle feeding by *An. arabiensis* in the irrigated villages	No	No	*An. arabiensis*: zoophilic	Mean tropical livestock units per village	Zooprophylaxis
Palsson et al. [35]	Guinea Bissau	30 households	Presence of pigs indoors associated with increased mosquito abundance (χ^2^ = 17.63, p < 0.001) but the presence of goats was not (χ^2^ = 1.08, p < 0.30). Goats were relatively uncommon compared to pigs (relative prevalence of livestock not reported)	No	No	*An. gambiae* s.l. (*An. gambiae* s.s. most abundant)	Presence of pigs or goats inside the house	Zoopotentiation
Temu et al. [41]	Mozambique	8,338 children from 2,748 households	Pig keeping associated with increased odds of malaria infection (OR 3.2, CI 2.1–4.9)	Yes	Yes	*An. gambiae* s.s.: anthropophilic, *An. funestus*: anthropophilic	Children living in households with chickens, goats, sheep, cows, pigs	Zoopotentiation
Tirados et al. [34]	Ethiopia	63,194 mosquitoes	HLC caught significantly more mosquitoes (163 mosquitoes/trap/night) than CBT (26 mosquitoes/trap/night, F = 35.9, p < 0.001) outdoors in areas of high cattle: human ratio compared to areas of low cattle: human ratio (HLC = 3.1, CBT = 2.1, no significant difference reported)	NA	NA	*An. arabiensis*: anthropophilic, exophagic	Cattle: human ratio 0.6:1 vs 17:1.	Zoopotentiation
Tirados et al.^a^ [22]	Ethiopia	Not reported	Outdoor HLC of *An. arabiensis* was not affected by the presence of a surrounding cattle ring, while the presence of a surrounding cattle ring reduced the outdoor HLC for *An. pharoensis* by 44% (p < 0.05). Indoor HLC did not differ from outdoors for either vector species. The indoor HLC decreased by 49% (p < 0.01) in presence of cattle ring for *An. arabiensis.* The catch of *An. arabiensis* in HBT was 25 times greater than in CBT (p < 0.001) whereas, for *An. pharoens*is there was no significant difference. HBT and CBT catches were unaffected by a ring of cattle for either vector species	NA	NA	*An. arabiensis*: opportunistic, exophagic	Presence of a ring of 20 cattle surrounding the place where a person was (either outside or inside hut)	Zooprophylaxis
Yamamoto et al. [43]	Burkina Faso	117 cases, 221 controls (women and children <9 years)	In univariable analyses, keeping donkeys (OR 0.59, CI 0.34–1.01), rabbits (OR 0.52, CI 0.25–1.09), and pigs (0.26, CI 0.07–0.89) within the compound had a significantly protective effect at the p < 0.20 level. While no effect was found for cows (OR 0.84, CI 0.45–1.54), sheep (OR 0.84, CI 0.51–1.37), goats (OR 0.08, CI 0.60–1.93), or poultry (OR 1.14, CI 0.68–1.90). No difference between malaria cases and controls associated with animal ownership after adjusting for bed net use and level of education (odds ratio of multivariate analysis not reported)	Yes	Yes^b^	*An. gambiae, An. funestus, An. arabiensis*	Animals kept in compound	None

*NA* not applicable due to nature of study design, *HBI* human blood index, *OBET* odor baited entry trap, *PSC* pyrethrum spray catch, *HLC* human landing catch, *HBT/CBT* human/cattle baited trap, *OR* odds ratio, *aRR* adjusted rate ratio, *CI* 95% confidence interval.

^a^Experimental design, observational design if not otherwise stated.

^b^Controlled for education level.

### Outcome measures

Four studies measured parasitaemia as an outcome. Three articles defined parasitaemia by positive identification of the parasite by thick and thin blood smears and one used a positive result on a malaria rapid diagnostic test (RDT). One study used recurrent household-level malaria infection defined as two or more infections for two or more household members over nine screening events but did not report the screening method used. Four studies reported mosquito feeding behaviour as measured by human blood index, which is the proportion of blood meals taken on a human out of the total number of blood meals taken. Five studies used mosquito abundance or mosquito presence as their outcome measure and four studies measured host attraction either by human landing catches or human-baited traps. One study reported both human blood index and mosquito abundance as outcome measures and another used both human blood index and host attractiveness by human landing catch.

### Key determinants of zooprophylaxis and zoopotentiation

Two main factors were consistently associated with zooprophylaxis and zoopotentiation: the predominant vector species present, and the location of livestock relative to humans, particularly during peak feeding times. Zooprophylaxis was considered to be dependent on the relative preference of mosquitoes for animal hosts (zoophily) in seven studies. Where the predominant mosquito species prefers human to animal hosts (anthropophily), and human hosts are available, keeping livestock nearby is unlikely to result in zooprophylaxis. Relative zoophily was reported as an important predictor of zooprophylaxis in five studies where multiple mosquito species were present. For example, *Anopheles gambiae* sensu stricto and *Anopheles funestus* were generally found to be anthropophilic compared to other species such as *Anopheles pharoensis* and *Anopheles arabiensis*, which were readily deferred from humans to feeding on livestock species [11, 13, 22, 33].

In some cases, *An. arabiensis* were found to be opportunistic in their host choices, or were anthropophilic but exophagic (prefer to feed outdoors) and therefore would feed on animals if no humans were found outdoors [34]. Many of the entomological studies [11, 12, 33, 34] collected only indoor resting mosquitoes for the assessment of blood meals, which may bias samples towards endophilic (indoor resting) and endophagic (indoor feeding) species, which often tend to be anthropophilic [24]. Mosquito species were not identified in the five studies that measured human malaria infection as the outcome.

Fourteen studies found that proximity or location of livestock relative to humans influenced malaria risk. When animals were housed inside at night, or in close proximity to sleeping rooms, malaria risk increased [11, 16, 23, 24, 33, 35, 36]. In contrast, when livestock were housed in separate shelters some distance away, malaria risk decreased [22, 34, 37]. However, some studies failed to find an association between location of livestock and zooprophylaxis or zoopotentiation. For example, in Lao PDR, owning a cow doubled the risk of mosquito house entry but keeping livestock near or underneath the house at night had no effect [38]. Similarly, a cohort study in The Gambia examined parasite prevalence in children sleeping within 20 m of the nearest cow compared to children sleeping at least 50 m from the nearest cow. No difference could be found in parasite prevalence between the groups when socio-economic factors were taken into account. It should be noted, however, that other livestock, such as goats, donkeys and horses, were commonly found in participating households but were not included in the analysis [10]. While no study specifically tested the impact of keeping livestock at varying distances on malaria risk, Maia and colleagues were unable to detect an effect of cattle at a distance of 20 m on human landing catches of mosquitoes [21].

Relative abundance of livestock to humans, or high cattle: human ratio may influence the success of zooprophylaxis [11, 39]. Three studies carried out in the Rift Valley of southern Ethiopia, where *An. arabiensis* is the main malaria vector, examined the relationship between cattle: human ratio and malaria risk. Two of these studies found no association [22, 34]. The third study did not account for the effect of humans sleeping on raised platforms in trees above cattle to avoid mosquito bites (with high cattle: human ratio) compared to the other two sites where humans slept in traditional dwellings (with lower cattle: human ratio) [12].

### Influence of modifying variables

Two contextual factors were shown to modify the association between malaria risk and livestock: the use of bed nets and socio-economic status. The use of bed nets seems to be an effect modifying factor, preventing even highly anthropophilic species from feeding on humans, forcing them to feed on livestock as an alternative [33]. While two studies found that bed nets had no impact on malaria infection [40], or mosquito house entry [38] and another reported that pig ownership remained a significant risk factor for positive RDT when bed nets were accounted for [41], six studies reported a relationship between bed nets and zooprophylaxis [8, 10, 11, 33, 42, 43]. In two of these studies, the effect of zooprophylaxis was diminished or became non-significant when bed net use was controlled for [10, 43]. Iwashita et al. reported that bed nets dramatically reduced human blood feeding in the presence of livestock [33]. A study conducted within a rice irrigation scheme in Kenya suggested that the cause of lower prevalence of malaria in villages where irrigation took place (and where prevalence was expected to be high) was a result of preferential feeding on livestock [8]. Bed net use was not measured in this study. Other work in the same location has suggested that bed net usage is promoted heavily in irrigated areas where malaria risk is known to be high [42].

Socio-economic status, measured as wealth or asset ownership was considered in four studies [10, 23, 38, 41]. One study identified a decrease in malaria prevalence with animal ownership, but controlling for wealth removed the effect of zooprophylaxis [10]. This study used a financial index based on livestock value to measure wealth and, therefore, collinearity might be expected between the presence of livestock and wealth. A second study noted that, in univariable analysis, sheep keeping was associated with decreased odds of infection with malaria while pig keeping was associated with increased odds of infection. When wealth was accounted for, the association with sheep ownership was no longer statistically significant while the relationship with pig ownership persisted [41]. Ghebreyesus et al. included household radio ownership in multivariable analysis and found that livestock sleeping inside the house increased incidence of infection in children [23]. Hiscox et al. did not find that household television ownership was significantly associated with mosquito house entry in univariable analysis, and it was therefore not included in multivariable analysis [38]. Yamamoto et al. controlled for maternal education level, a robust and commonly used measure of socio-economic status [44], and found that the protective effect of donkeys, rabbits and pigs was removed when level of education and bed net use were controlled for [43]. These studies and others [8] emphasized the strong association between measures of socio-economic status and malaria risk. This important association can confound the relationship between animal ownership and malaria prevalence given that animal ownership is a reflection of social standing. Socio-economic status is likely an important unmeasured confounder affecting zooprophylaxis in the scientific evidence base.

## Discussion

This systematic realist review points to three key findings regarding the context under which zooprophylaxis may be utilized as a component of IVM. First, zooprophylaxis is most likely to be effective when the mosquito species present do not have a strong preference for human hosts. Second, in order to take advantage of mosquito preference for animals, animals must be kept out of human sleeping quarters at night. There is evidence that even in the context of mosquito species with preference for animal hosts, close proximity to humans at night may result in zoopotentiation. Third, where bed nets are used, mosquitoes are more likely to feed on animal hosts as an alternative.

Proximity of livestock to humans at night has been identified as an important factor in zooprophylaxis [45]. What remains unclear is the appropriate distance at which livestock should be kept in order to promote zooprophylaxis or prevent zoopotentiation. It is also unknown whether this distance differs between regions, species and contexts. Incidence rates of *Plasmodium vivax* were reduced in Sri Lankan households where cattle sheds were located within 70 m of the home when wealth, bed nets and other protective measures were considered; however, this effect was weak (RR 0.70, 95% CI 0.47–1.03) [46]. Current evidence supports the exclusion of animals from human dwellings at night, particularly where mosquito species are zoophilic. Improved estimation and precision around appropriate livestock proximities would benefit from the inclusion of livestock species, their number and location, and use of bed nets or other malaria prophylaxis in future studies.

Mosquito species characteristics were also identified as a key predictor of zooprophylaxis and zoopotentiation. Highly anthropophilic species were generally unaffected by the presence or absence of livestock whereas zoophilic and opportunistic species may be deterred from humans in the presence of alternative hosts. This is consistent with a model by Saul predicting that for vectors with a low human biting index, an increase in animal host density can significantly decrease disease transmission, while the same did not hold for weakly zoophilic species [18]. Similarly, Franco et al. predict that in the presence of moderately zoophilic vectors, such as *An. arabiensis*, the introduction of livestock would increase malaria transmission except in two cases: (1) where vector carrying capacity has already been reached in the system and the addition of livestock hosts does not increase vector density; and, (2) where livestock density and availability are so great as to counteract the effect of increased vector density associated with the introduction of livestock [47].

With regard to the impact of bed nets, since the rate of disease transmission is dependent upon host species interaction, any intervention that decreases contact between host and vector will decrease the risk of infection [5]. This has been corroborated by mathematical transmission models which find that while increased cattle density can decrease malaria transmission when sufficient animals are present and are housed separately, the most successful reduction transmission occurs when personal protective measures are also employed [18, 39]. Where accessibility of humans relative to animals is decreased, it is predicted that malaria prevalence and number of bites will decrease [18, 47]. Time of biting and human behaviour may also have an impact on the effectiveness of bed nets. If people are outdoors during peak biting times, bed nets will not provide protection against mosquito bites [21].

Socio-economic factors may be important unmeasured confounders in studies of zooprophylaxis. Risk factors for malaria are related to poverty through limited access to preventative measures such as bed nets, screened windows, closed roofing, and adequate health care [48]. Livestock ownership is also associated with increased socio-economic status, especially among the rural poor [49–51]. It has been suggested that in addition to zooprophylactic effects, livestock may be a confounder for reduced malaria risk as those who own livestock may also be able to afford preventative and treatment measures [10] or have better overall health and nutritional status [51, 52]. Households keeping animals indoors at night may represent those who are financially restricted from providing alternative livestock shelters, further complicating the inter-relationships between wealth and animal ownership in malaria transmission.

## Conclusions

There is scientific evidence to support zooprophylaxis where the dominant vector is highly zoophilic and livestock are kept away from human sleeping quarters during peak vector activity. The use of protection such as bed nets may be complementary, and would be expected to reduce the measured effect of zooprophylaxis in empirical studies. Where vector preference is mixed, varied or unknown, or where the appropriate distance of livestock from sleeping quarters is in debate, there is insufficient evidence to support the use of zooprophylaxis, and some evidence to suggest the possibility of zoopotentiation. Research in three priority areas is required for clearer evidence of contexts to maximize the likelihood of zooprophylaxis and minimize the likelihood of zoopotentiation: (1) estimation of the distance threshold and conditions whereby processes of zoopotentiation transition to zooprophylaxis for specific livestock host and mosquito vector species combinations; (2) consideration of the preference of species to feed indoors *versus* outdoors in entomologic studies in order to accurately assess mosquito host preferences; and, (3) inclusion of socio-economic factors and the use of other prophylactic measures as key covariates in empirical research assessing zooprophylaxis and zoopotentiation. These research priorities may aid in the development of guidelines for the use of zooprophylaxis as a malaria control intervention for agricultural extension agencies who may wish to make livestock management recommendations, such as the optimal placement of livestock shelters with respect to human sleeping quarters. Zooprophylaxis has the potential to contribute to IVM strategies due to its non-chemical nature, optimal combination with bed nets, potential social desirability, and minimal financial requirements. It will require interdisciplinary collaboration between agricultural extension officers, veterinarians and health care professionals with ongoing monitoring of efficacy.

## Additional file

Additional file 1: Articles excluded from review and reasons for exclusion.
