# Supplementary material for: A systematic, realist review of zooprophylaxis for malaria control
Source: Malar J. 2015 Aug 12;14:313. doi: 10.1186/s12936-015-0822-0 (PMC4533963; doi:10.1186/s12936-015-0822-0)
Supplement: Additional file 1: — Articles excluded from review and reasons for exclusion. [file 12936_2015_822_MOESM1_ESM.pdf]

Supplementary material: articles excluded from review and reasons for exclusion

Stage	Reason for exclusion	Article	Total	Remaining
Stage 1: Document retrieval				
	Non-scientific literature. Source type: patent	Lee DW, Zooprophylaxis vaccinating apparatus for inoculating vaccine to domestic animal such as pig, is provided with press bayonet lock for firing capsule to domestic animal.	1	
Total excluded at stage 1			**1**	75
Stage 2: Title review				
	Non-malarial disease	Cruz-Pacheco et al., 2012. Control Measures for Chagas Disease. MATHEMATICAL BIOSCIENCES.		
		Kasili et al., 2009. Comparative attractiveness of CO2-baited CDC light traps and animal baits to *Phlebotomus duboscqi* sandflies. JOURNAL OF VECTOR-BORNE DISEASES.		
		Chelbi et al., 2008. Zooprophylaxis: Impact of Breeding Rabbits Around Houses on Reducing the Indoor Abundance of *Phlebotomus papatasi*. VECTOR BORNE AND ZOONOTIC DISEASES.		
		Kirby et al., 2008. Risk factors for house-entry by culicine mosquitoes in a rural town and satellite villages in The Gambia. PARASITES AND VECTORS.		
		Achukwi et al., 2007. Successful vaccination against *Onchocerca ochengi* infestation in cattle using live *Onchocerca volvulus* infective larvae. PARASITE IMMUNOLOGY.		
		Seidenfaden et al., 2001. Combined benefits of annual mass treatment with ivermectin and cattle zooprophylaxis on the severity of human onchocerciasis in northern Cameroon. TROPICAL MEDICINE AND INTERNATIONAL HEALTH.		
		Vasquez et al., 1999. Effects of non-susceptible hosts on the infection with *Trypanosoma cruzi* of the vector *Triatoma infestans*: an experimental model. MEMORIAS DO INSTITUTO OSWALDO CRUZ		
		Cecere et al., 1997. Effects of chickens on the prevalence of infestation and population density of *Triatoma infestans* in rural houses of north-west Argentina. MEDICAL AND VETERINARY ENTOMOLOGY.		
		Gurtler et al., 1997. Shifting host choices of the vector of Chagas disease, *Triatoma infestans*, in relation to the availability of hosts in houses in north-west Argentina. JOURNAL OF APPLIED ECOLOGY.		
		Mutinga et al., 1990. Epidemiology of leishmaniases in Kenya – natural host preference of wild caught phlebotomine sandflies in Baringo District, Kenya. EAST AFRICAN MEDICAL JOURNAL.		
		Hess & Hayes, 1970. Relative potential of domestic animals for zooprophylaxis against mosquito vectors of encephalitis. AMERICAN JOURNAL OF TROPICAL MEDICINE AND HYGIENE.		
		Nelson et al., 1968. Studies on heterologous immunity in schistosomiasis. 1. Heterologous schistosome immunity in mice. BULLETIN OF THE WORLD HEALTH ORGANIZATION.		
		Amin et al., 1968. Studies on heterologous immunity in schistosomiasis. 2. Heterologous schistosome immunity in Rhesus monkeys. BULLETIN OF THE WORLD HEALTH ORGANIZATION.		
		Sim et al., 1987. Human in vitro immune-reactions to animal filariids. TROPICAL MEDICINE AND PARASITOLOGY.		

		Gurtler et al., 2014. Domestic Animals Hosts Strongly Influence Human-Feeding Rates of the Chagas Disease Vector *Triatoma infestans* in Argentina. PLOS NEGLECTED TROPICAL DISEASES.		
		Kaabi & Ahmed, 2013. Assessing the effect of zooprophylaxis on zoonotic cutaneous leishmaniasis transmission: A system dynamics approach. BIOSYSTEMS.		
		Richter et al., 2013. Spatial stratification of various Lyme disease spirochetes in a Central European site. FEMS MICROBIOLOGY ECOLOGY.		
			17	
	Mathematical model	Hassanali et al., 2008. Zooprophylactic diversion of mosquitoes from human to alternative hosts: A static simulation model. ECOLOGICAL MODELING.		
		Kelly and Thompson, 2000. Epidemiology and optimal foraging: modelling the ideal free distribution of insect vectors. PARASITOLOGY.		
		Sota & Mogi, 1989. Effectiveness of zooprophylaxis in malaria control – a theoretical inquiry, with a model for mosquito populations with 2 bloodmeal hosts. MEDICAL AND VETERINARY ENTOMOLOGY.		
			3	
	Editorial/other	Rowland et al., 1996. 'O come, let us wallow...' A reply - Muddying the waters, or turning zooprophylaxis from failure to success. TRANSACTIONS OF THE ROYAL SOCIETY OF TROPICAL MEDICINE AND HYGIENE.		
		Iliev T, 1981. Pure and applied scientific work of Prof. G. Pavlov in the field of zooprophylaxis. VETERINARNO-MEDITSINKI NAUKI.		
			2	
	Review	Service, MW, 1991. Agricultural development and arthropod-borne diseases: A review. REVISTA DE SAUDE PUBLICA.		
			1	
Total excluded at Stage 2			**23**	52
Stage 3: Abstract review				
	Mathematical model	Nah et al., 2010. The dilution effect of the domestic animal population on the transmission of *P. vivax* malaria. JOURNAL OF THEORETICAL BIOLOGY.		
		Le Menach et al., 2005. The unexpected importance of mosquito oviposition behaviour for malaria: non-productive larval habitats can be sources for malaria transmission. MALARIA JOURNAL.		
		Killeen et al., 2004. Rationalizing historical successes of malaria control in Africa in terms of mosquito resource availability management. AMERICAN JOURNAL OF TROPICAL MEDICINE AND HYGIENE.		
		Kawaguchi et al., 2004. Combining zooprophylaxis and insecticide spraying: a malaria-control strategy limiting the development of insecticide resistance in vector mosquitoes. PROCEEDINGS OF THE ROYAL SOCIETY B-BIOLOGICAL SCIENCES.		
		Saul A, 2003. Zooprophylaxis or zoopotentiation: the outcome of introducing animals on vector transmission is highly dependent on the mosquito mortality while searching. MALARIA JOURNAL.		
		Franco et al., 2014. Controlling Malaria Using Livestock-Based Interventions: A One Health Approach. PLOS ONE.		
			6	
	Review	Randolph et al., 2012. Pangloss revisited: a critique of the dilution effect and the biodiversity-buffers-disease paradigm. PARASITOLOGY.		
		Bettini & Romi, 1998. Zooprophylaxis: old and new problems. PARASSITOLOGIA.		

		AULT, SK, 1994. Environmental management – A reemerging vector control strategy. AMERICAN JOURNAL OF TROPICAL MEDICINE AND HYGIENE.		
		Lacey & Lacey, 1990. The medical importance of riceland mosquitoes and their control using alternatives to chemical insecticides. JOURNAL OF THE AMERICAN MOSQUITO CONTROL ASSOCIATION. Supplement.		
		Goriup, S, 1989. Analysis of available measures for malaria control in Africa south of the Sahara. TRANSACTIONS OF THE ROYAL SOCIETY OF TROPICAL MEDICINE AND HYGIENE.		
			5	
	Editorial/other	Kweka et al., 2008. Impact of *Anopheles arabiensis* feeding and resting behaviour in zooprophylaxis for malaria control. AMERICAN JOURNAL OF TROPICAL MEDICINE AND HYGIENE.		
		Dobson et al., 2006. Sacred Cows and Sympathetic Squirrels: The Importance of Biological Diversity to Human Health. PLOS MEDICINE		
		Charlwood, D, 2001. Zooprophylaxis: are we in Plato's cave? TRENDS IN PARASITOLOGY.		
			3	
	Non-malarial disease	Basanez et al., 2007. Density-dependent host choice by disease vectors: epidemiological implications of the ideal free distribution. TRANSACTIONS OF THE ROYAL SOCIETY OF TROPICAL MEDICINE AND HYGIENE.		
		Renz et al., 1994. Cattle, worms and zooprophylaxis. PARASITE-JOURNAL DE LA SOCIETE FRANÇAISE DE PARASITOLOGIE.		
			2	
	Non-malaria specific (febrile illness)	Deressa et al., 2007. Household and socioeconomic factors associated with childhood febrile illnesses and treatment seeking behaviour in an area of epidemic malaria in rural Ethiopia. TRANSACTIONS OF THE ROYAL SOCIETY OF TROPICAL MEDICINE AND HYGIENE.		
			1	
	Research framework	Gu et al., 2008. Habitat-based larval interventions: A new perspective for malaria control. AMERICAN JOURNAL OF TROPICAL MEDICINE AND HYGIENE.		
			1	
Total excluded at Stage 3			**18**	34
Stage 4: Full article review				
	Non-malaria specific (all cause child mortality)	Komazawa et al., 2012. Are Long-Lasting Insecticidal Nets Effective for Preventing Childhood Deaths among Non-Net Users? A Community-Based Cohort Study in Western Kenya. PLOS ONE.		
	No measure of human malaria risk or exposure	Lyimo et al., 2012. Does cattle melieu provide a potential point to target wild exophilic *Anophelies arabiensis* (Diptera: Culicidae) with entomopathogenic fungus? A bioinsecticide zooprophylaxis strategy for vector control. JOURNAL OF PARASITOLOGY RESEARCH.		
	Inappropriate measure of livestock effect.	Bugoro et al., 2011. Bionomics of the malaria vector *Anopheles farauti* in Temotu Province, Solomon Islands: issues for malaria elimination. MALARIA JOURNAL.		
	Livestock exposure measured at the village level but effect on malaria risk not investigated.	Muturi et al., 2008. Effect of Rice Cultivation on Malaria Transmission in Central Kenya. AMERICAN JOURNAL OF TROPICAL MEDICINE AND HYGIENE.		
	Mosquito mortality as outcome measure, not human malaria risk or exposure.	Mahande et al., 2007. Role of cattle treated with deltamethrine in areas with a high population of *Anopheles arabiensis* in Moshi, Northern Tanzania. MALARIA JOURNAL.		

	Impact of livestock on malaria risk not investigated	Gopaul, R, 1995. Surveillance entomologique à Maurice. CAHIERS SANTÉ.		
	Review	Rasnitsyn & Lebedeva, 1995. The prospects of the use of attractants for the mosquito control (Diptera, Culicidae). ZOOLOGICHESKY ZHURNAL		
	No statistical analysis	Kirnowordoyo & Supalin, 1985. Zooprophylaxis as a useful tool for control of *A. aconitus* transmitted malaria in Central Java, Indonesia. JOURNAL OF COMMUNICABLE DISEASE.		
	No measure of human malaria risk or exposure	Naz et al., 2013. Efficacy of Ivermectin for Control of Zoophilic Malaria Vectors in Pakistan. PAKISTAN JOURNAL OF ZOOLOGY.		
	Unable to draw conclusions based on results presented	Kaburi et al., 2009. Effects of long-lasting insecticidal nets and zooprophylaxis on mosquito feeding behaviour and density in Mwea, central Kenya. JOURNAL OF VECTOR-BORNE DISEASES.		
	No control/comparison group	Bhatt et al., 2008. Dynamics of *Anopheles culidifacies* -transmitted malaria in the absence of effective zooprophylaxis in a riverine settlement in Gujarat, India. CURRENT SCIENCE.		
	No statistical analysis with regards to outcomes of interest to this review	Mahande et al., 2007. Feeding and resting behaviour of malaria vector, *Anopheles arabiensis* with reference to zooprophylaxis. MALARIA JOURNAL.		
	Sample size	McCall et al., 2001. Evidence for memorized site-fidelity in *Anopheles arabiensis*. TRANSACTIONS OF THE ROYAL SOCIETY OF TROPICAL MEDICINE AND HYGIENE.		
	Livestock exposure not measured	Muriu et al., 2008. Host choice and multiple blood feeding behaviour of malaria vectors and other anophelines in Mwea rice scheme, Kenya. MALARIA JOURNAL.		
Total excluded at stage 4			14	20
